# Cardioprotective Effect of Resveratrol in a Postinfarction Heart Failure Model

**DOI:** 10.1155/2017/6819281

**Published:** 2017-10-03

**Authors:** Adam Riba, Laszlo Deres, Balazs Sumegi, Kalman Toth, Eszter Szabados, Robert Halmosi

**Affiliations:** ^1^1st Department of Medicine, University of Pécs Medical school, Pécs, Hungary; ^2^Szentagothai Research Centre, University of Pécs, Pécs, Hungary; ^3^Department of Biochemistry and Medical Chemistry, University of Pécs Medical school, Pécs, Hungary; ^4^MTA-PTE Nuclear-Mitochondrial Interactions Research Group, Pécs, Hungary

## Abstract

Despite great advances in therapies observed during the last decades, heart failure (HF) remained a major health problem in western countries. In order to further improve symptoms and survival in patients with heart failure, novel therapeutic strategies are needed. In some animal models of HF resveratrol (RES), it was able to prevent cardiac hypertrophy, contractile dysfunction, and remodeling. Several molecular mechanisms are thought to be involved in its protective effects, such as inhibition of prohypertrophic signaling molecules, improvement of myocardial Ca^2+^ handling, regulation of autophagy, and the reduction of oxidative stress and inflammation. In our present study, we wished to further examine the effects of RES on prosurvival (Akt-1, GSK-3*β*) and stress signaling (p38-MAPK, ERK 1/2, and MKP-1) pathways, on oxidative stress (iNOS, COX-2 activity, and ROS formation), and ultimately on left ventricular function, hypertrophy and fibrosis in a murine, and isoproterenol- (ISO-) induced postinfarction heart failure model. RES treatment improved left ventricle function, decreased interstitial fibrosis, cardiac hypertrophy, and the level of plasma BNP induced by ISO treatment. ISO also increased the activation of P38-MAPK, ERK1/2^Thr183-Tyr185^, COX-2, iNOS, and ROS formation and decreased the phosphorylation of Akt-1, GSK-3*β*, and MKP-1, which were favorably influenced by RES. According to our results, regulation of these pathways may also contribute to the beneficial effects of RES in HF.

## 1. Introduction

Despite significant advances in therapy, heart failure (HF) is a constantly growing medical and social burden in western societies. Pharmacological inhibition of the RAAS and the adrenergic system resulted in substantial reduction in mortality [1]. However, further blocking of the neuroendocrine axis has failed to fulfill our hopes, so new ideas and approaches are needed in the treatment of heart failure. Oxidative stress is thought to play an important role in different cardiac pathological conditions such as I/R injury, fibrosis, cardiac hypertrophy, remodeling, and heart failure [2]. Excessive ROS may cause extensive oxidative damage to proteins, DNA, and lipids resulting in damaged cardiomyocyte functions including contractility, ion transport, and calcium cycling [3]. Moreover, ROS induce different pathological intracellular signaling pathways ultimately evoking apoptosis and necrosis. Regulation of intracellular ROS formation and modification of the stress responding signaling pathways may prevent or slow pathological processes in HF [4].

**Figure 1:**
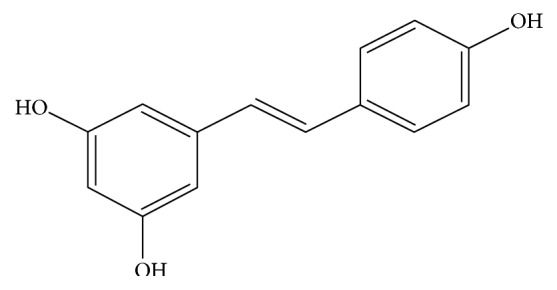
Chemical structure of resveratrol (RES).

Resveratrol (3,5,4′-trihydroxystilbene) (RES) (Figure 1) is a natural phytoalexin found in a wide variety of plant species including grapes and nuts and present in varying concentration in red wines [5]. Numerous experimental studies have verified that RES interferes with several pathological processes in different cardiovascular diseases such as myocardial ischemia [6], myocarditis [7], cardiac hypertrophy [8], and heart failure [4]. Multiple mechanisms have been proposed to be responsible for the protective effects of RES in HF, including reducing oxidative stress and inflammation [9, 10], inhibiting pathological hypertrophic signaling [11], improving Ca^2+^ handling [12], decreasing apoptosis, and modifying autophagy through different intracellular pathways [13].

The aim of the present study was to further examine RES in a postinfarction heart failure animal model, where isoproterenol, a strong sympathetic agent, was used to induce myocardial infarction, causing patchy, predominantly subendocardial necrosis and fibrosis [14]. We examined the effect of RES on left ventricular function, myocyte hypertrophy, collagen deposition, ROS production, and intracellular signaling pathways taking part in the process of cardiac remodeling and heart failure.

## 2. Methods

### 2.1. Animals

Male 20-week-old Wistar rats (410–480 g) were used for the experiments. The experiment was approved by the Animal Research Review Committee of the University of Pécs Medical School (Permit number: BA02/2000–2/2010), and the animals received care according to the Guide for the Care and Use of Laboratory Animals published by the US National Institute of Health (NIH Publication Number 85–23, revised 1996). The animals were housed under standardized conditions, 12 h dark-light cycle in solid-bottomed polypropylene cages, and received commercial rat chew ad libitum. Resveratrol of herbal origin (with >98% t-resveratrol content) was purchased from Argina Nutraceuticals (Fót, Hungary). Ethanol was used to prepare a stock solution of RES which was added to 30 ml of drinking water. We got a clean solution without any undissolved residue. Dosage of RES was set to 15 mg/kg/day. After finishing this first 30 ml of drinking water, the animals got clear water for the rest of the day [15].

### 2.2. Experimental Protocol

The rats were treated twice on two consecutive days with 80 mg/kg ISO (Sigma-Aldrich) or vehicle (physiological saline solution) subcutaneously to induce postinfarction remodeling as previously described. The animals were divided into four groups: control group (C) received clear water without ISO treatment; the second group of rats received two subcutaneous injections of isoproterenol at the dosage of 80 mg/kg (ISO); ISO + RES group (ISO + RES) received resveratrol with ISO treatment; and the resveratrol group (RES) received resveratrol without ISO treatment. RES inhibitor treatment was delayed 24 h to avoid suppression of infarct size. At the end of the 8-week-long period, body weights were measured, animals were sacrificed, and the hearts were removed. Atria and great vessels were trimmed from the ventricles; the weight of the ventricles was measured and normalized to body mass. Afterward, ventricles were fixed in 10% formalin for histology or freeze clamped for Western blot analysis [16].

### 2.3. Noninvasive Evaluation of Cardiac Structure and Function

Transthoracic echocardiography was performed under inhalation anesthesia at the beginning of the experiment and on the day of sacrifice. The rats were lightly anesthetized with a mixture of 1.5% isoflurane and 98.5% oxygen. The chest of the animals was shaved and acoustic coupling gel was applied. The animals were imaged in the left lateral position, and a warming pad was used to maintain normothermia. Cardiac diameter and functions were measured from short and long axis views at the midpapillary level using a VEVO 770 high-resolution ultrasound imaging system (VisualSonics, Toronto, Canada), which is equipped with a 25-MHz transducer. The investigators were blinded to the treatment protocol. Left ventricular (LV), ejection fraction (EF), LV end-diastolic volume, LV end-systolic volume, and the thickness of the septum and posterior wall were determined. EF (percentage) was calculated by 100 × [(LVEDV−LVESV)/LVEDV] [16, 17].

### 2.4. Histology

For histologic examination, ventricles were fixed in formalin and sliced and embedded in paraffin. Sections (5 *μ*m thick) were cut serially from the base to apex. LV sections were stained with Masson's trichrome to detect interstitial fibrosis and quantified by the NIH ImageJ image processing program via its color deconvolution plug-in [17].

### 2.5. Nitrotyrosine Immunohistochemical Staining

We performed immunohistochemical staining for nitrotyrosine, a nitro-oxidative stress marker, using a previously described method. Extensively stained areas were also quantified using the NIH ImageJ image processing program via its color deconvolution plug-in [18].

### 2.6. Determination of Plasma B-Type Natriuretic Peptide Level

Blood samples were collected into Vacutainer tubes containing EDTA and aprotinin (0.6 IU/ml) and centrifuged at 1600*g* for 15 minutes at 4°C to obtain plasma, which was collected and kept at −70°C. Plasma B-type natriuretic peptide-45 levels (BNP-45) were determined by enzyme immunoassay method (BNP-45, Rat EIA Kit, Phoenix Pharmaceuticals Inc., CA, USA) [17].

### 2.7. Western Blot Analysis

Fifty milligrams of heart samples were homogenized in ice-cold Tris buffer (50 mmol/l, pH 8.0) containing 50 mM sodium vanadate and protease inhibitor cocktail (Sigma-Aldrich Co., Budapest, Hungary) and harvested in 2x concentrated SDS-polyacrylamide gel electrophoresis sample buffer. To ensure the same protein concentration in each well, protein levels were measured with Nanodrop. GAPDH was used only as a representative loading control. Proteins were separated on 12% SDS-polyacrylamide gel and transferred to nitrocellulose membranes. After blocking (2 h with 3% nonfat milk in Tris-buffered saline), membranes were probed overnight at 4°C with antibodies recognizing the following antigens: phosphospecific mitogen-activated protein (MAP) kinase phosphatase-1 (*MKP*-*1*) Ser359 (1 : 1000), phosphospecific Akt-1/protein kinase B-*α* Ser473 (1 : 1000), phosphospecific glycogen synthase kinase (GSK)-3*β* Ser9 (1 : 1000), and phosphospecific p38 mitogen-activated protein kinase (p38-MAPK) Thr180-Gly-Tyr182 (1 : 1000), ERK1/2^Thr183-Tyr185^, COX-2 (1 : 1000), and iNOS (1 : 1000). Membranes were washed six times for 5 min in Tris-buffered saline (pH 7.5) containing 0.2% Tween (TBST) before the addition of goat anti-rabbit horseradish peroxidase-conjugated secondary antibody (1 : 3000 dilution; Bio-Rad, Budapest, Hungary). Membranes were washed six times for 5 min in TBST, and the antibody-antigen complexes were visualized by means of enhanced chemiluminescence. The results of Western blots were quantified using the NIH ImageJ program.

### 2.8. Statistical Analysis

Statistical analysis was performed by analysis of variance, and all of the data were expressed as the mean ± SEM. The homogeneity of the groups was tested by *F*-test (Levene's test). There were no significant differences among the groups. Comparisons among groups were made by one-way ANOVA followed by Bonferroni correction or Tukey HSD's post hoc tests in SPSS for Windows, version 21.0. All data are expressed as mean ± SEM. A value of *P* < 0.05 was considered statistically significant.

## 3. Results

### 3.1. Resveratrol Treatment Improved the Gravimetric Parameters in ISO-Induced Heart Failure Model

Body weights did not differ significantly among the four groups at the beginning or the end of the experiment. Gravimetric measurements were performed and significantly elevated ventricular weight (WV, g) as well as ventricular weight normalized to body weight (WV/BW, mg/g) (C versus ISO, *P* < 0.05) and to tibia length (TL) (WV/TL, mg/mm) (C versus ISO, *P* < 0.05) were detected. Resveratrol treatment prevented the unfavorable changes in gravimetric parameters indicating hypertrophy in the ISO + RES group (ISO + RES versus ISO, *P* < 0.05) (Table 1).

### 3.2. Resveratrol Decreased the Heart Failure-Induced Elevation of Plasma BNP Level

**Figure 2:**
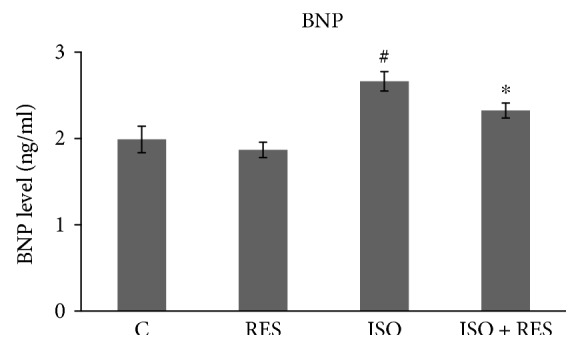
RES inhibited the heart failure-induced elevation of plasma BNP level. Plasma BNP level was determined using an ELISA method as described in Methods. C: control animals; RES: animals treated with resveratrol for 8 weeks; ISO: animals 8 weeks after ISO administration; ISO + RES: animals treated with resveratrol 8 weeks after ISO administration. Values are mean ± SEM, ^∗^*P* < 0.05 versus ISO, and ^#^*P* < 0.05 versus C.

ISO administration led to a significant increase in BNP level in the ISO group 8 weeks after myocardial infarction in our study (C versus ISO, *P* < 0.05). However, resveratrol significantly attenuated this response (ISO + RES versus ISO, *P* < 0.05) suggesting that resveratrol decreases the severity of postinfarction heart failure. There was no significant difference between the C and the RES groups (Figure 2).

### 3.3. Resveratrol Improved Left Ventricular Function and Moderated Left Ventricular Hypertrophy in ISO-Treated Rats

**Figure 3:**
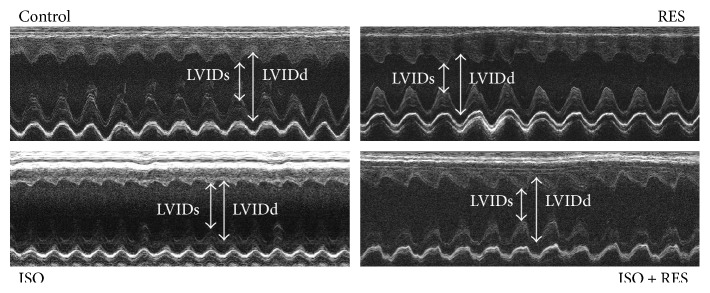
Representative echocardiographic M-mode images of left ventricles of animals from control, RES, ISO, and ISO + RES groups.

The echocardiographic parameters of the animals did not differ significantly from each other at the beginning of the study. Heart rate did not differ significantly during anesthesia among the groups. LVESV and LVIDs were significantly higher in the ISO group (ISO versus C and ISO versus RES *P* < 0.05). The thickness of the septum and posterior wall and calculated LV mass were also higher in the ISO group (indicating the presence of ventricular hypertrophy) compared to the control group (ISO versus C, *P* < 0.05). Resveratrol treatment significantly reduced these unfavorable alterations. Systolic left ventricular function (EF %) was significantly lower in the ISO group (ISO versus C, *P* < 0.05), and this deterioration was significantly improved by resveratrol administration (ISO versus ISO + RES, *P* < 0.05) (Table 2, Figure 3).

### 3.4. Resveratrol Decreased Interstitial Collagen Deposition in the Myocardium

**Figure 4:**
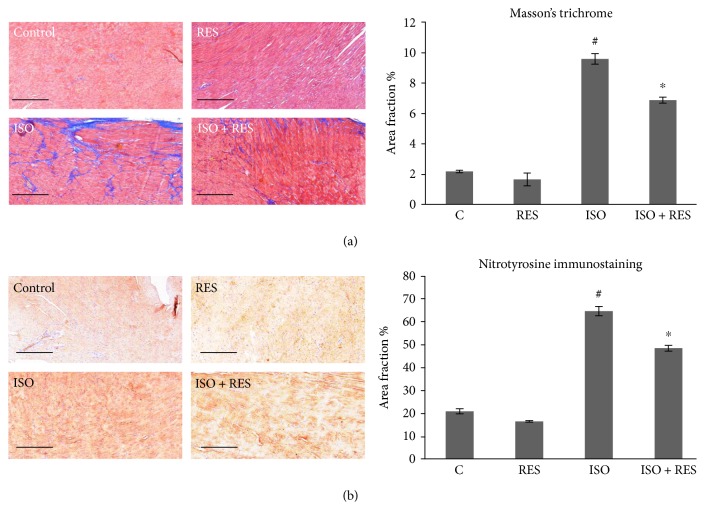
RES treatment moderated ISO-induced interstitial collagen deposition and protein nitrosylation in ISO-induced heart failure. (a) Representative sections stained with Masson's trichrome, scale bar: 500 *μ*m, and magnification is10-fold. Control: age-matched control rats. RES: age-matched animals treated with resveratrol for 8 weeks. ISO: age-matched animals 8 weeks after ISO administration. ISO + RES: age-matched animals treated with resveratrol, 8 weeks after ISO administration. Values are mean ± SEM, *P* < 0.05 (ISO versus control group), and *P* < 0.05 (ISO + RES versus ISO group). (b) Representative immunohistochemical stainings for nitrotyrosine (NT, brown staining, scale bar: 500 *μ*m, and 10x magnification) in the myocardium of the following: control: age-matched control rats; RES: age-matched animals treated with resveratrol for 8 weeks; ISO: age-matched animals 8 weeks after ISO administration; and ISO + RES: age-matched animals treated with resveratrol, 8 weeks after ISO administration. Values are mean ± SEM, ^#^*P* < 0.05 (ISO versus control group), and ^∗^*P* < 0.05 (ISO + RES versus ISO group).

Marked scar tissue formation was revealed by histological analysis after ISO stress in failing rat hearts compared to the control group (*P* < 0.05). Resveratrol treatment significantly decreased the extent of interstitial fibrosis (*P* < 0.05). Resveratrol alone did not cause any significant alterations in physiological conditions related to myocardial hypertrophy or interstitial collagen deposition (Figure 4(a)).

### 3.5. Effects of Resveratrol on the Oxidative Stress Marker Nitrotyrosine

The presence of oxidative stress was confirmed in our rodent heart failure model by the measurement of NT, which is a product of tyrosine nitration. Almost no immunostaining for NT was observed in myocardial sections of the control group. In contrast, in animals with heart failure (ISO), immunostaining was significantly more extensive (*P* < 0.05, C versus ISO), but this increase was attenuated by RES treatment (*P* < 0.05, ISO versus ISO + RES; Figure 4(b)).

### 3.6. Resveratrol Favorably Influenced the Phosphorylation of Akt-1^Ser473^ and GSK-3*β*^Ser9^ in Failing Myocardium

**Figure 5:**
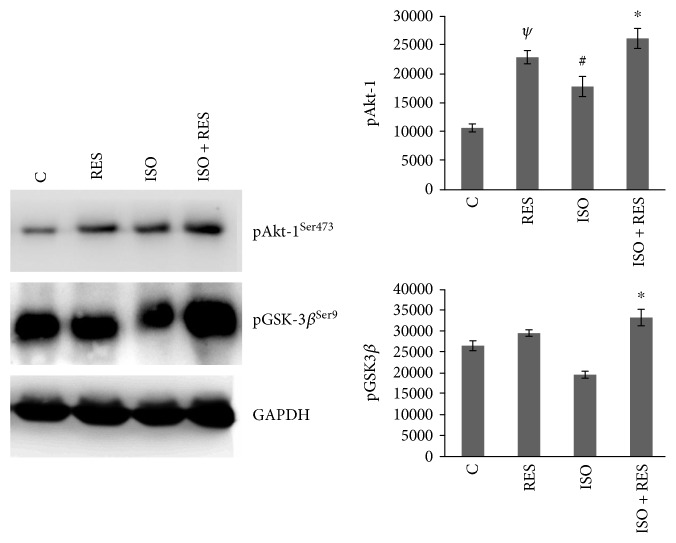
Effect of resveratrol treatment on Akt-1^Ser473^ and GSK-3*β*^Ser9^. Representative Western blot analysis of Akt-1^Ser473^, GSK-3*β*^Ser9^, and p38-MAPK^Thr180-Gly-Tyr182^ and phosphorylation and densitometric evaluation are shown. GAPDH was used as a loading control. Representative blots and bar diagrams of three independent experiments are presented. C: control animals; RES: animals treated with resveratrol for 8 weeks; ISO: animals 8 weeks after ISO administration; ISO + RES: animals treated with resveratrol, 8 weeks after ISO administration. Values are mean ± SEM, ^#^*P* < 0.05 versus control, ^∗^*P* < 0.05 versus ISO, and ^*ψ*^*P* < 0.05 (C versus RES).

The level of Akt-1^Ser473^ was significantly higher in the RES and ISO + RES groups compared to the control and ISO groups (*P* < 0.05), respectively. Although the level of Akt-1^Ser473^ was significantly elevated in ISO-treated animals (ISO versus C *P* < 0.05), the highest activities were observed in the ISO + RES group (ISO versus ISO + RES *P* < 0.05). The level of GSK-3*β*^Ser9^ was also slightly elevated in the RES group compared to the control group and significantly decreased in ISO animals compared to control animals (*P* < 0.05, C versus ISO). The elevation in the ISO + RES group was significant compared to the ISO group too (*P* < 0.05, ISO + RES versus ISO). GAPDH was used as a loading control (Figure 5).

### 3.7. Resveratrol Attenuated the Phosphorylation of p38-MAPK^Thr180-Gly-Tyr182^ and ERK1/2^Thr183-Tyr185^ and Increased the Amount of MKP-1 in ISO-Stressed Hearts

**Figure 6:**
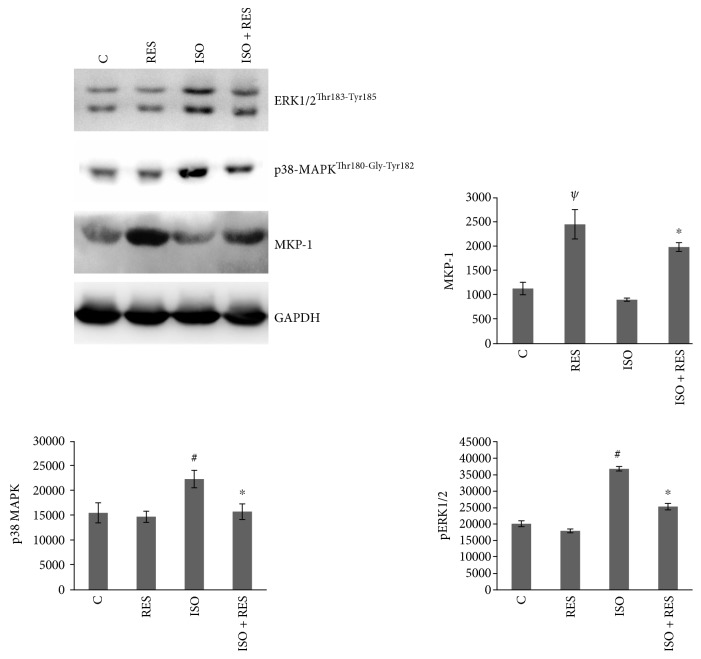
Effect of resveratrol treatment on the phosphorylation of p38-MAPK^Thr180-Gly-Tyr182^, ERK1/2^Thr183-Tyr185^, and on the amount of MKP-1. Representative Western blot analysis of p38-MAPK^Thr180-Gly-Tyr182^, ERK1/2 phosphorylation, and MKP-1 level; a densitometric evaluation is shown. GAPDH was used as a loading control. Representative blots and bar diagrams of three independent experiments are presented. C: control animals; RES: animals treated with resveratrol for 8 weeks; ISO: animals 8 weeks after ISO administration; ISO + RES: animals treated with resveratrol, 8 weeks after ISO administration. Values are mean ± SEM, ^#^*P* < 0.05 (control versus ISO), ^*ψ*^*P* < 0.01 (C versus RES), and ^∗^*P* < 0.05 (ISO versus ISO + RES).

The level of phosphorylation of p38-MAPK^Thr180-Gly-Tyr182^ and ERK1/2^Thr183-Tyr185^ was significantly elevated in the ISO group compared to the control group (C versus ISO *P* < 0.05). There was a significant reduction in the phosphorylation level of p38-MAPK^Thr180-Gly-Tyr182^ in the ISO + RES group versus the ISO group (*P* < 0.05, ISO versus ISO + RES). The activation of ERK1/2^Thr183-Tyr185^ was also significantly reduced in the ISO + RES group compared to the ISO group (*P* < 0.05, ISO versus ISO + RES). Consequently, the steady state amount of MKP-1 was significantly elevated in the ISO + RES group compared to the ISO group. Interestingly, there was a strong and significant elevation in the RES group compared to the control group (*P* < 0.05, C versus RES). GAPDH was used as a loading control (Figure 6).

### 3.8. Resveratrol Decreased the Expression of COX-2 and iNOS

**Figure 7:**
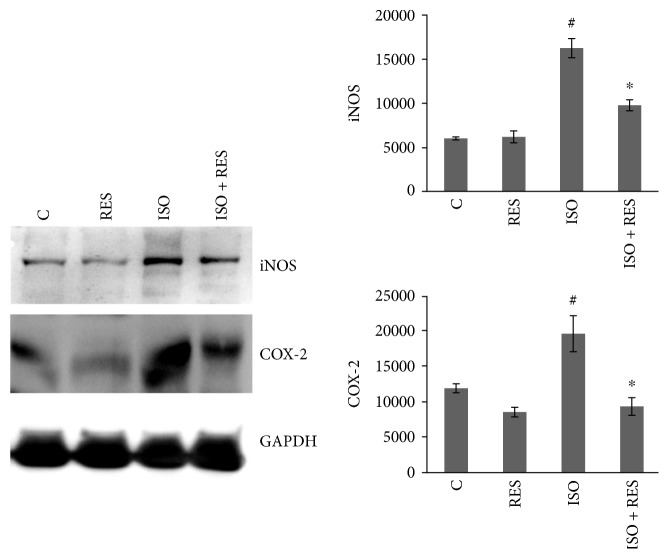
Effect of resveratrol treatment on COX-2 and iNOS. Representative Western blot analysis of COX-2 and iNOS activation and densitometric evaluation is shown. GAPDH was used as a loading control. Representative blots and bar diagrams of three independent experiments are presented. C: control animals; RES: animals treated with resveratrol for 8 weeks; ISO: animals 8 weeks after ISO administration; ISO + RES: animals treated with resveratrol, 8 weeks after ISO administration. Values are mean ± SEM, ^#^*P* < 0.05 (control versus ISO), and ^∗^*P* < 0.05 (ISO versus ISO + RES).

The expression of COX-2 and iNOS was significantly elevated in ISO compared to the control (*P* < 0.05, C versus ISO) and significantly decreased in ISO + RES compared to ISO (*P* < 0.05, ISO versus ISO + RES). Although the activation level of COX-2 was markedly decreased in RES compared to the control, the only significant difference was between ISO + RES and ISO. GAPDH was used as a loading control (Figure 7).

## 4. Discussion

Chakraborty et al. examined the protective effect of resveratrol and atorvastatin and their combination on isoproterenol-induced cardiac hypertrophy in rats. They found that RES pretreatment was able to decrease infarct size and myocardial necroenzyme level and restored myocardial endogenous antioxidant level [19]. Moreover, our working group has previously demonstrated the beneficial effects of an alcohol-free red wine extract in an isoproterenol-induced heart failure model [8].

This time, we aimed to test the cardioprotective effect of RES on oxidative stress and different signaling pathways in an advanced stage of heart failure. Subcutaneous administration of ISO produces patchy myocardial necrosis with subsequent hypertrophy, fibrosis, and remodeling leading to heart failure similar to that observed in patients after myocardial infarction [20]. Plasma BNP levels (Figure 2), left ventricular wall thickness, and dimensions were increased and the systolic left ventricular function was significantly decreased in ISO-treated animals. RES was capable of preserving LV function and moderated the severity of heart failure (Table 2). Excessive collagen deposition is the main histological characteristic of ventricular remodeling. The damaged myocardium is replaced by scar tissue stabilizing the ventricular wall but leading to systolic and diastolic dysfunction and arrhythmias. In our study, RES prevented the marked fibrosis induced by ISO treatment (Figure 4(a)).

There is a large amount of evidence that ROS release plays a pivotal role in the development of heart failure [21]. We observed increased immunohistochemical staining against nitrotyrosine (NT)—a marker of oxidative stress—on ISO-treated animal heart samples, which was significantly decreased by RES treatment (Figure 4(b)) indicating that RES was able to reduce myocardial ROS production. This antioxidant property of RES was investigated many times in different heart failure models including hypertensive [22], pressure overload [23, 24], myocarditis [7], or chemotherapy induced [25, 26] and genetic models of HF [27].

Excessive ROS formation induces different intracellular signaling pathways regulating cardiac remodeling, myocyte survival, apoptosis, and necrosis [28]. Here, we investigated the effect of RES on ERK 1/2, p38-MAPK, Akt-1, and GSK-3*β* pathways which plays a critical role in cardiac hypertrophy and ventricular dilatation [29], myocyte survival, apoptosis, autophagy, and necrosis [29–31]. Akt activation inhibits cardiomyocyte apoptosis and improves surviving of cardiomyocytes in the ischemic heart [32]. Akt exerts its protective effect through phosphorylation of the Bcl-2 family and GSK-3*β* [33]. Akt-1 is a key molecule in the signaling of physiological hypertrophy, and it has a pivotal role in the prevention of pathological cardiac growth [29]. Interestingly, in our investigation, phosphorylation of Akt-1 and GSK-3*β* was elevated in all treated groups compared to the control group and the highest level of phosphorylation was in the ISO + RES group (Figure 5), indicating that RES presumably further augments the stress ISO-induced activity of endogenous prosurvival signaling pathways. Xi et al. examined the effect of RES against reperfusion injury on an isolated rat heart, and they also found that the cardioprotective effect of RES involved the increased phosphorylation of GSK-3*β* [11].

There are conflicting data in the literature regarding the role of MAP kinases in the regulation of cell survival [29, 30, 34–37]. A wide variety of extra and intracellular stress signals can induce sequential phosphorylation and activation of MAPK kinases via phosphorylation on both threonine and tyrosine residues [31]. The effect of RES on the activation of MAPK cascades was previously investigated on cardiomyocytes. Becatti et al. found that RES protected cardiomyocytes from oxidative injury by SIRT1 overexpression by reducing p38-MAPK activity [38]. Lv and Zhou demonstrated that RES attenuated cell death and apoptosis induced by oxidative stress by upregulating autophagy via inhibiting the p38 MAPK pathway [39]. In an animal study by Gao et al., they found that RES ameliorated diabetes-induced cardiac dysfunction through the AT1R-ERK/p38 MAPK signaling pathway [40]. We investigated the phosphorylation (activation) of p38-MAPK^Thr180-Gly-Tyr182^ and ERK1/2^Thr183-Tyr185^ which was elevated in ISO-treated groups but RES significantly decreased their activation (Figure 6). These changes were probably due to the increased production of MAPK phosphatase-1 (MKP-1) which is the major regulator of MAPKs [31, 37, 41]. In accordance with this, the amount of MKP-1 was increased in the RES-treated groups compared to untreated animals (Figure 6).

Whereas COX-1 plays a housekeeping role, COX-2 plays a major part in inflammation, atherosclerosis, and tumor formation [42–44]. Previous studies showed that COX-2 is upregulated by p38-MAPK and ERK1/2 [45]. Moreover, prolonged activation of COX-2 produces cardiac cell death, leading to gradual loss of myocardial function, and eventually heart failure. The effect of RES on COX-2 activity was not investigated previously neither on cardiomyocytes nor on a heart failure animal model. We found in our postinfarction animal model that RES was able to reduce the activation of COX-2 induced by ISO treatment (Figure 7).

Previous in vivo animal and human studies demonstrated the elevated expression of iNOS in heart failure [46]. Overexpression of iNOS in the myocardium of mice resulted in peroxynitrite generation [47–50], which was also detected in our model and it has also been demonstrated that elevated nitrotyrosine formation results in an increase in iNOS levels, creating a vicious circle of harmful effects [51]. Such evidence indicates that iNOS is also a factor, in addition to COX-2, in the development of postinfarction heart failure [52]. It has been shown that iNOS, as well as COX-2 expression level, is strongly correlated with the phosphorylation level of the MAPKs (p38-MAPK and ERK1/2^Thr183-Tyr185^) [53–55]. Also, it has been reported that flavonoids like quercetin, galangin, and apigenin can downregulate iNOS expression by modulating enzyme activity related to signal transduction [56, 57]. In our study, the ISO-enhanced expression of iNOS was reduced by RES treatment (Figure 7) indicating suppressed free radical formation.

## 5. Conclusion

The protective effects of the nutritional agent RES against the development of postinfarction heart failure were investigated in a murine model. RES improved the left ventricular function and decreased myocardial hypertrophy, fibrosis, and the severity of heart failure. Moreover, RES decreased the oxidative stress and favorably modified the activity of several intracellular signaling pathways including Akt-1, GSK-3*β*, p38-MAPK, ERK1/2, MKP-1, COX-2, and iNOS. According to our results, regulation of these pathways may also contribute to the beneficial effects of RES in HF.

## Figures and Tables

**Table 1:** Effects of RES and ISO on the gravimetric parameters.

Group	Control	RES	ISO	ISO + RES
Weight (g)	595.86 ± 15.15	596.00 ± 21.30	544.50 ± 11.63	593.86 ± 18.41
Ventricular weight (g)	1.33 ± 0.01	1.31 ± 0.01	1.53 ± 0.02^#^	1.35 ± 0.01^∗^
Tibia length (mm)	51.43 ± 0.72	51.57 ± 0.72	50.86 ± 0.55	49.86 ± 0.35
Ventricular weight/body weight (mg/g)	2.25 ± 0.06	2.21 ± 0.08	2.81 ± 0.06^#^	2.29 ± 0.09^∗^
Ventricular weight/tibia length (mg/mm)	26.03 ± 0.47	25.33 ± 0.33	29.96 ± 0.28^#^	27.02 ± 0.13^∗^

Eight weeks after ISO-induced myocardial infarction, body weight, mass of ventricles, and tibia length were measured. Ventricular weight/body weight (mg/g) and ventricular weight/tibia length (mg/mm) ratios were calculated. The results are expressed as mean ± SEM. ^#^*P* < 0.05 versus control. ^∗^*P* < 0.05 versus ISO.

**Table 2:** Resveratrol improved left ventricular function in ISO-treated rats and reduced left ventricular hypertrophy.

	Baseline	C	RES	ISO	ISO + RES
EF (%)	75.62 ± 0.87	71.70 ± 1.61	72.47 ± 1.69	56.96 ± 1.43^#^	67.49 ± 1.14^∗^
Septum (mm)	1.63 ± 0.05	1.65 ± 0.10	1.61 ± 0.03	1.82 ± 0.03^#^	1.70 ± 0.02^∗^
PW (mm)	1.57 ± 0.03	1.59 ± 0.07	1.59 ± 0.03	1.81 ± 0.06^#^	1.60 ± 0.02^∗^
LVIDd (mm)	8.19 ± 0.11	8.44 ± 0.22	8.43 ± 0.17	7.88 ± 0.12	8.41 ± 0.23
LVIDs (mm)	4.42 ± 0.08	4.85 ± 0.09	4.69 ± 0.19	5.70 ± 0.2^#^	4.90 ± 0.12^∗^
LVEDV (*μ*l)	364.23 ± 10.38	393.36 ± 19.32	386.40 ± 16.82	365.54 ± 6.64	401.59 ± 18.63
LVESV (*μ*l)	88.83 ± 4.40	109.9 ± 4.53	106.14 ± 7.26	157.71 ± 7.29^#^	130.27 ± 6.69^∗^
LV mass (mg)	994.1 ± 21.8	1035.31 ± 59.79	1038.38 ± 44.44	1239.14 ± 76.5^#^	1041.85 ± 35.50^∗^

C: control group; RES: resveratrol group; ISO: isoproterenol-treated group; ISO + RES: ISO + resveratrol group. EF: ejection fraction; LVESV: left ventricular end-systolic volume; LVEDV: left ventricular end-diastolic volume; LVIDd: diastolic left ventricular inner diameter; LVIDs: systolic left-ventricular inner diameter. The results are expressed as mean ± SEM. ^#^*P* < 0.05 versus control. ^∗^*P* < 0.05 versus ISO.

## Abbreviations

RES: Resveratrol (3,5,4′-trihydroxystilbene)
ISO: Isoproterenol
LV: Left ventricular
VW: Ventricular weight
BW: Body weight
TL: Tibia length
EF: Ejection fraction
LVEDV: LV end-diastolic volume
LVESV: LV end-systolic volume
LVIDd: Diastolic left ventricular inner diameter
LVIDs: Systolic left-ventricular inner diameter
BNP: B-type natriuretic peptide
NT: Nitrotyrosine.
